# Aligning brain tumor imaging: generating synthetic 3D FA maps from T1-weighted MRI using CycleGAN models

**DOI:** 10.1093/bjrai/ubag019

**Published:** 2026-09-25

**Authors:** Xin Du, Francesca M Cozzi, Stephen J Price, Rajesh Jena

**Affiliations:** The Cavendish Laboratory, University of Cambridge, Cambridge, CB3 0HE, United Kingdom; Cambridge Brain Tumor Imaging Lab, University of Cambridge, Cambridge, CB2 1TN, United Kingdom; Department of Clinical Neurosciences, University of Cambridge, Cambridge, CB2 1TN, United Kingdom; Department of Oncology, University of Cambridge, Cambridge, CB2 1TN, United Kingdom

**Keywords:** CycleGAN model, FA maps, T1-weighted MRI, tumor-affected

## Abstract

**Objectives:**

This study addresses the issue of spatial misalignment between fractional anisotropy (FA) maps and tractography atlases in neuroimaging. We propose a CycleGAN-based method to generate FA and directionally encoded color (DEC) maps directly from T1-weighted MRI scans, applicable to both healthy and tumor-affected cases.

**Methods:**

A CycleGAN model was developed and trained using unpaired data to convert T1-weighted MRI images into FA and DEC maps. The quality of the generated maps was assessed using Structural Similarity Index (SSIM) and Peak Signal-to-Noise Ratio (PSNR) metrics. Special emphasis was placed on evaluating performance in tumor regions. Radiological assessments were also conducted to determine the clinical applicability of the generated maps.

**Results:**

The CycleGAN-based model effectively generated high-fidelity FA and DEC maps, with robust SSIM and PSNR values, particularly in tumor regions. Radiological evaluations highlighted the potential of this approach to enhance clinical workflows, offering a reliable AI-driven alternative to traditional image processing methods.

**Conclusions:**

This CycleGAN-based approach successfully mitigates the problem of spatial misalignment by generating accurate FA and DEC maps from T1-weighted MRI scans. The method offers a promising pathway for integrating assessments of white matter integrity into predictive models without requiring additional imaging.

**Advances in knowledge:**

This study introduces the first use of CycleGAN for generating FA and DEC maps from T1-weighted images for tumor-affected cases, presenting a method that could enhance neuroimaging procedures by reducing the need for additional scans and improving efficiency in clinical settings.

## Introduction

Diffusion MRI has become a mainstay of medical neuroimaging research. In particular, diffusion tensor imaging (DTI) and other derivative images, such as fractional anisotropy (FA) and directionally encoded color (DEC) maps, are increasingly integrated into research pipelines. DTI is an advanced MRI modality that incorporates diffusion-sensitizing magnetic field gradients during image acquisition; these added gradients make the captured signal sensitive to the directional movement of water molecules in the brain^1^ [this technique iterates over multiple diffusion gradient directions to create a 3D diffusion tensor model, represented as a 3 × 3 symmetric matrix that describes water diffusion directionality per voxel (a 3D pixel)]. This directional water movement allows for the mapping of white matter tracts, aiding in assessing their integrity and detecting abnormalities. It also provides insights into brain connectivity, revealing how different regions are linked, which is essential for understanding neural networks.^2^ Additionally, abnormal diffusion patterns can indicate pathological changes, potentiating the early diagnosis of neurological conditions. Overall, these diffusion patterns elucidate important characteristics of brain structure and function in both healthy and disease states.

Fractional anisotropy, which reflects the directionality of brain fibers, is the most widely used metric derived from DTI, owing to its sensitivity to microstructural changes in the axonal fibers of white matter pathways.^2^ This metric is particularly valuable in the context of tumors, as certain brain neoplasms, such as gliomas, are known to infiltrate and migrate along these white matter tracts.^3–11^ Moreover, FA has shown promise as a predictive marker for glioblastoma recurrence.^12–14^ These insights are instrumental for advancing current and future efforts to optimize radiotherapy margins for this aggressive and deadly tumor.

Furthermore, DECs provide a color representation of the principal diffusion direction along the predominant fiber orientation [red corresponds to the medial-lateral (left-right) orientation; green to the anterior-posterior (front-back) orientation; and blue to the superior-inferior (up-down) orientation].^15^ In the context of neurosurgical planning for resection of brain tumors, these DEC maps offer valuable insight into the relationship between white matter tracts and tumor lesions.^16^ The DEC is related to the FA image, in that the intensity of the displayed colors on the DEC map is modulated by FA values.^15^

While FA maps are crucial for assessing white matter microstructural integrity and playing a fundamental role in structural connectivity analysis and tractography-based models, the integration of FA maps into analytical pipelines is often hindered by spatial misalignment between standard FA maps and tractography atlases, leading to challenges in cross-modal data fusion that limit their broader applicability.^17–22^ Additionally, the acquisition time for MRI and DTI sequences in one session is approximately 45 minutes, whereas the patient must attempt to remain still within the magnet for this duration of time. Inevitably, however, patients shift, and over time, the position of the patient relative to the first image capture degrades, leading to spatial misalignment between images taken at the beginning of the study and those taken at the end. While these misalignments are not problematic for standard radiological interpretations, they do become troublesome when attempting to fuse images and conduct multimodal imaging analysis.

To address this limitation, generating FA maps directly from T1-weighted images (T1WI) presents a promising alternative. T1WI scans are widely available in routine clinical practice and offer high-resolution anatomical details, making them an ideal candidate for synthesizing diffusion-derived metrics. Recent advances in artificial intelligence (AI) have enabled deep learning models to generate high-fidelity medical images, enhancing diagnostic capabilities across multiple modalities, including MRI and CT.^23^ AI-driven generative models, such as deep learning-based image translation networks, have demonstrated the ability to synthesize missing imaging modalities with high accuracy, improving image quality while reducing dependency on specialized sequences.^24–26^ These advancements are particularly beneficial for patients who may not have access to diffusion-weighted imaging (DWI) due to scanning constraints or contraindications, such as the presence of implanted medical devices, renal insufficiency, or sensitivity to contrast agents.

In this study, we propose using a CycleGAN framework^27^ to generate 3D FA and DEC maps directly from T1WI scans for both healthy brains and tumor-affected brains. Unlike traditional deep learning models that require perfectly paired training data, CycleGAN leverages unpaired image-to-image translation, making it particularly suitable for medical imaging applications where annotated datasets are often scarce and different modalities are challenging to register perfectly. By synthesizing FA maps from T1WI directly, our approach ensures spatial consistency with atlases, facilitating seamless integration into predictive models while enhancing the diagnostic utility of conventional MRI scans.

This study aims to demonstrate the feasibility of AI-driven 3D FA synthesis, showing that it can preserve high-fidelity representations of white matter structures and pathological regions such as tumors. Incorporating this method into clinical workflows has the potential to broaden access to diffusion-based biomarkers, improve neuroimaging analysis, and ultimately enhance patient care by reducing the need for additional, time-consuming imaging sequences.

## Related work

The application of advanced machine learning models for cross-modality generation in medical imaging has gained significant attention.^28^ CycleGAN, emerging as a prominent approach introduced by Zhu et al.,^27^ allows for the learning of mappings between unpaired medical image datasets, making it particularly useful in scenarios where paired training samples are not feasible. Numerous studies have highlighted the efficiency of CycleGAN in the transformation of medical images between different modalities.^29–31^

For example, Gong et al.^32^ exploited CycleGAN enhanced with channel-wise attention mechanisms to better focus on relevant features within MRI data while simultaneously constraining the structural similarity between synthetic and target CT images. This dual approach effectively integrates the rich contextual information from MRI and ensures that the generated CT images retain high fidelity. This approach minimizes the need for CT scans, thus reducing patient exposure to radiation and the burden associated with multiple imaging sessions and enhancing treatment planning in head and neck radiotherapy. Similarly, Wolterink et al.^33^ applied CycleGAN to synthesize MRI images from CT scans, offering supplemental diagnostic information while reducing overall scanning time and resource utilization.^34^

Furthermore, Gu et al.^35^ explored the application of CycleGAN in estimating FA values from T1WIs using a 2D framework on only healthy cases, illustrating the potential of these approaches to provide reliable assessments of white matter integrity without the necessity of diffusion-weighted imaging. These studies collectively demonstrate the transformative impact of CycleGAN in the medical imaging domain, paving the way for innovative diagnostic tools and efficient clinical workflows.

Beyond CycleGAN, style transfer models have also demonstrated potential in image generation. The method proposed by Huang and Belongie^36^ uses style representations to combine content from one modality with the style of another. Similarly, Cao et al.^37^ introduces StyleMapper, a medical image style transfer method that efficiently transforms breast MRI scans into unseen styles using limited training data. By utilizing a varied set of simulated medical imaging styles, StyleMapper enhances computational efficiency and enables arbitrary style transfer, which is crucial for managing different imaging protocols and scanner models in medical imaging. This method helps create a unified style dataset of medical images, improving training for downstream tasks like classification and object detection.

Diffusion models have recently emerged as a powerful alternative for generative tasks, including image synthesis. These models, which work by gradually denoising a random signal to create coherent images, have shown capability in generating high-quality medical images.^38^^,^^39^ Kim and Park^40^ introduce a model based on the latent diffusion model for image-to-image translation in 3D medical images, overcoming challenges in acquiring multi-modal images due to cost and safety concerns. Utilizing a switchable block architecture, the model generates high-quality 3D images without patch cropping and addresses limitations of 2D methods. The multiple switchable spatially adaptive normalization block enables various translation tasks with a single model.

These advancements underscore the versatility of various model architectures in tackling challenges in medical imaging, particularly in enhancing accessibility and alleviating patient burden. However, there is a scarcity of studies focused on generating 3D FA maps from normal MRI scans for both healthy and tumor-affected brains, overcoming the shortcoming of the missing out-of-slice information in 2D generation methods. Generating 3D FA maps is crucial for subsequent clinical research due to the complexities involved in spatial alignment and registration of FA maps to template scans. Our work leverages the capabilities of CycleGAN to address this challenge, offering a solution that minimizes the need for additional scan processing beyond routine procedures, thereby reducing the burden on patients.

## Datasets

Two datasets are used for synthetic image generation. For healthy cases, T1WI are sourced from the Human Connectome Project (HCP),^41^^,^^42^ comprising 1065 subjects. Following the settings of Gu et al.,^35^ 1000 randomly selected subjects are used for model training, while the remaining 65 serve as unseen test cases. The ground truth FA maps are obtained via diffusion tensor fitting and MAP-MRI fitting, as introduced by Gu et al.^35^

For tumor-affected cases, both pre-contrast T1WI and ground truth FA maps are sourced from the University of California San Francisco Preoperative Diffuse Glioma MRI (UCSF-PDGM) dataset,^43^ acquired using 3T scanners, similar to the healthy cases. This dataset consists of 501 patients, with 445 randomly assigned to the training set and 56 to the test set. The dataset includes glioma cases of 3 grades: Grade 2, Grade 3, and Grade 4. Further statistical details can be found in Calabrese et al.^43^

## Methods

### CycleGAN architecture

CycleGAN^27^ facilitates unpaired image-to-image translation by leveraging adversarial training and cycle consistency. The architecture consists of 2 generators, G:X→Y and F:Y→X, along with 2 discriminators, $D_{X}$ and $D_{Y}$, which distinguish between real and generated images in each domain, as shown in Figure 1.

**Figure 1:**
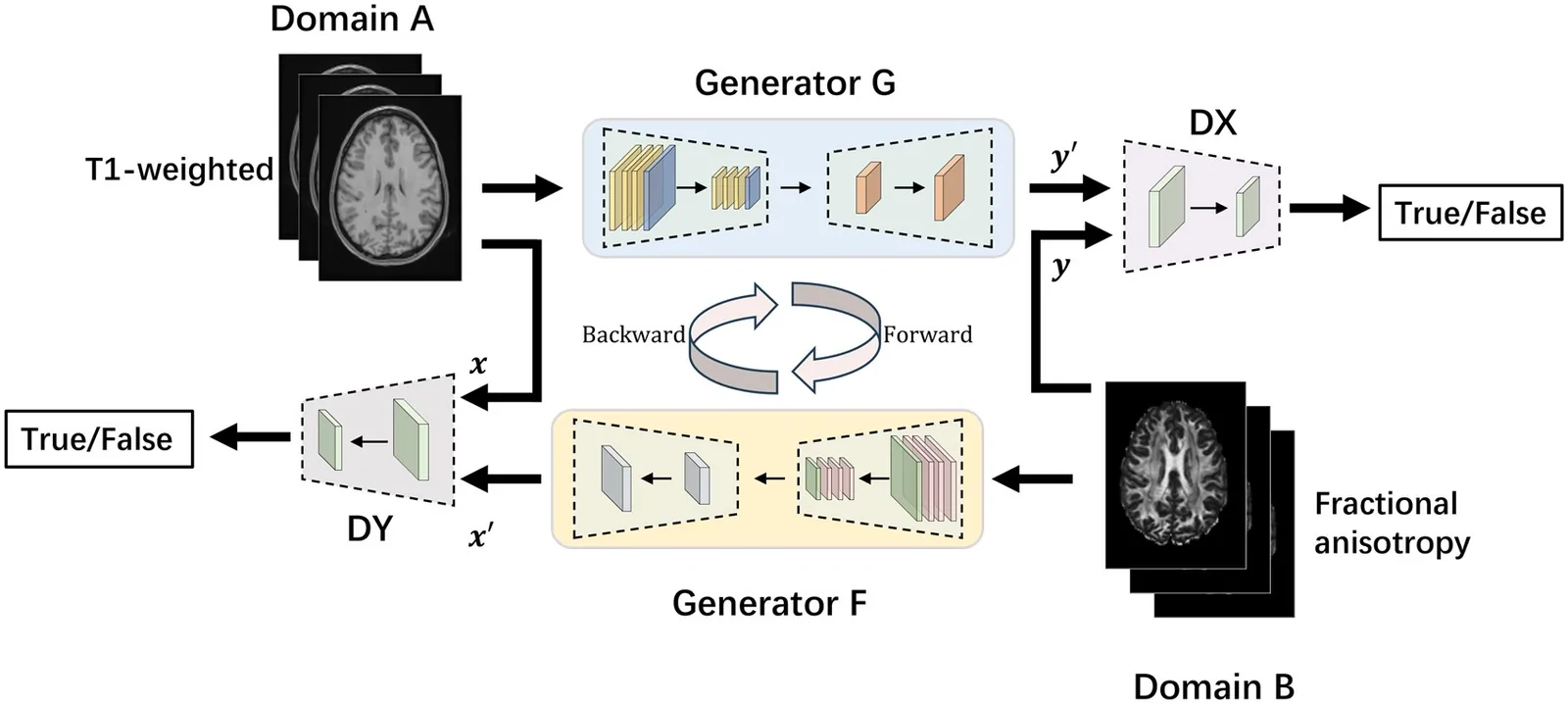
The schematic of 3D CycleGAN architecture.

Following the network architecture design of CycleGAN,^27^^,^^35^^,^^44–46^ a 3D cross-modality medical image generation model is constructed. Specifically, each generator consists of 3 convolutional layers (128×128), 9 residual blocks (256×256), and 2 fractionally-strided convolutional layers. Similar to the configuration proposed by Johnson et al.,^46^ instance normalization is employed. Each discriminator is composed of 5 convolutional layers with LeakyReLU activation and instance normalization. For generating FA maps, both the input and output channels are set to 1, whereas for DEC map generation, 3 channels are used.

### Adversarial loss

To ensure the generated images are indistinguishable from real images, CycleGAN employs adversarial loss for both mappings. For the X→Y translation, the objective function is defined as:


(1) $$\begin{array}{c} L_{\mathrm{GAN}}(G,D_{Y},X,Y)=E_{y}[\text{log}\;D_{Y}(y)]\\ +E_{x}[\text{log}(1-D_{Y}(G(x)))] \end{array}$$

A similar adversarial loss is applied for the Y→X mapping.

### Cycle consistency loss

Since unpaired training data lacks direct supervision, cycle consistency is enforced to ensure G(F(y))≈y and F(G(x))≈x. The cycle consistency loss is formulated as:


(2) $$\begin{array}{c} L_{\mathrm{cycle}}(G,F)=E_{x}[\|F(G(x))-x{\|}_{1}]\\ +E_{y}[\|G(F(y))-y{\|}_{1}] \end{array}$$

This constraint preserves content and structure during domain translation.

### Correlation coefficient loss

The standard CycleGAN relies on cycle-consistency and adversarial losses to enforce bidirectional domain mapping. However, the cycle-consistency loss operates on absolute intensity values and does not explicitly preserve local structural patterns, which is limiting in medical image synthesis where anatomical spatial relationships are critical.

Following the setting given by Ge et al.,^45^ the correlation coefficient (Cor-Coe) Loss addresses this gap by measuring similarity in terms of pixel or feature correlation rather than intensity magnitude. By maximizing the linear correlation between generated and real images, it captures structural similarity in a manner ie, invariant to global intensity shifts and scaling. This makes it particularly suited for cross-modal translation, where source and target domains exhibit fundamentally different intensity distributions. Additionally, by evaluating local correlation patterns, the Cor-Coe Loss encourages preservation of fine-grained anatomical details, such as white matter tract orientations in DEC maps, that pixel-wise or adversarial losses alone may not adequately capture.

For tumor-bearing brains, this property is especially valuable, as the Cor-Coe Loss maintains spatial coherence across healthy and pathological regions without imposing rigid intensity constraints that could suppress heterogeneous signal characteristics associated with tumor infiltration.


(3) $$L_{\text{Cor-Coe}}(G,F)=\frac{\text{Cov}(G(x),x)}{\sigma_{G}(x)\sigma_{x}}+\frac{\text{Cov}(F(y),y)}{\sigma_{F}(y)\sigma_{y}}$$

### Full objective function

The overall objective function combines adversarial loss, cycle consistency loss, and cor-coe loss:


(4) $$\begin{array}{c} L(G,F,D_{X},D_{Y})=L_{\mathrm{GAN}}(G,D_{Y},X,Y)\\ +L_{\mathrm{GAN}}(F,D_{X},Y,X)\\ +\lambda L_{\mathrm{cycle}}(G,F)\\ +\beta L_{\text{Cor-Coe}}(G,F) \end{array}$$

where λ controls the weight of the cycle consistency loss and β controls the weight of cor-coe loss. They are all set to 1.

### Training details

CycleGAN is trained using the Adam optimizer with a learning rate of 0.0002 and momentum parameters $\beta_{1}=0.5,\beta_{2}=0.999$. A batch size of 1 or 8 is typically used, depending on memory constraints. The model is trained for 200 epochs, with the first half using a constant learning rate and the latter half employing a linearly decaying learning rate. To improve training stability, least-squares loss is used for adversarial training instead of binary cross-entropy. All inputs are resized to 128-128-64. Notably, the choice of optimizer and its hyperparameters plays a critical role in the convergence of complex deep learning architectures; metaheuristic algorithms have been shown to effectively optimize such parameters, improving model convergence and overall performance [?].

For healthy brains, all models are trained from scratch with random initialization. For tumor cases, both training from scratch and transfer learning are applied. In transfer learning, the model is first pre-trained on healthy cases and then fine-tuned on tumor-affected cases to leverage prior knowledge from healthy brains and assess the impact of knowledge transfer on the generated images.

### Evaluation metrics

To assess the quality of the generated images, we employ 3 widely used image similarity metrics: Structural Similarity Index (SSIM), Multi-Scale Structural Similarity Index (MS-SSIM), and Peak Signal-to-Noise Ratio (PSNR), which collectively capture both pixel-level accuracy (PSNR) and perceptual similarity (SSIM and MS-SSIM). Additionally, 2 radiologists performed a visual assessment to distinguish between the generated and ground truth maps using a custom-developed tool. A screenshot of this software is shown in Figure 2.

**Figure 2:**
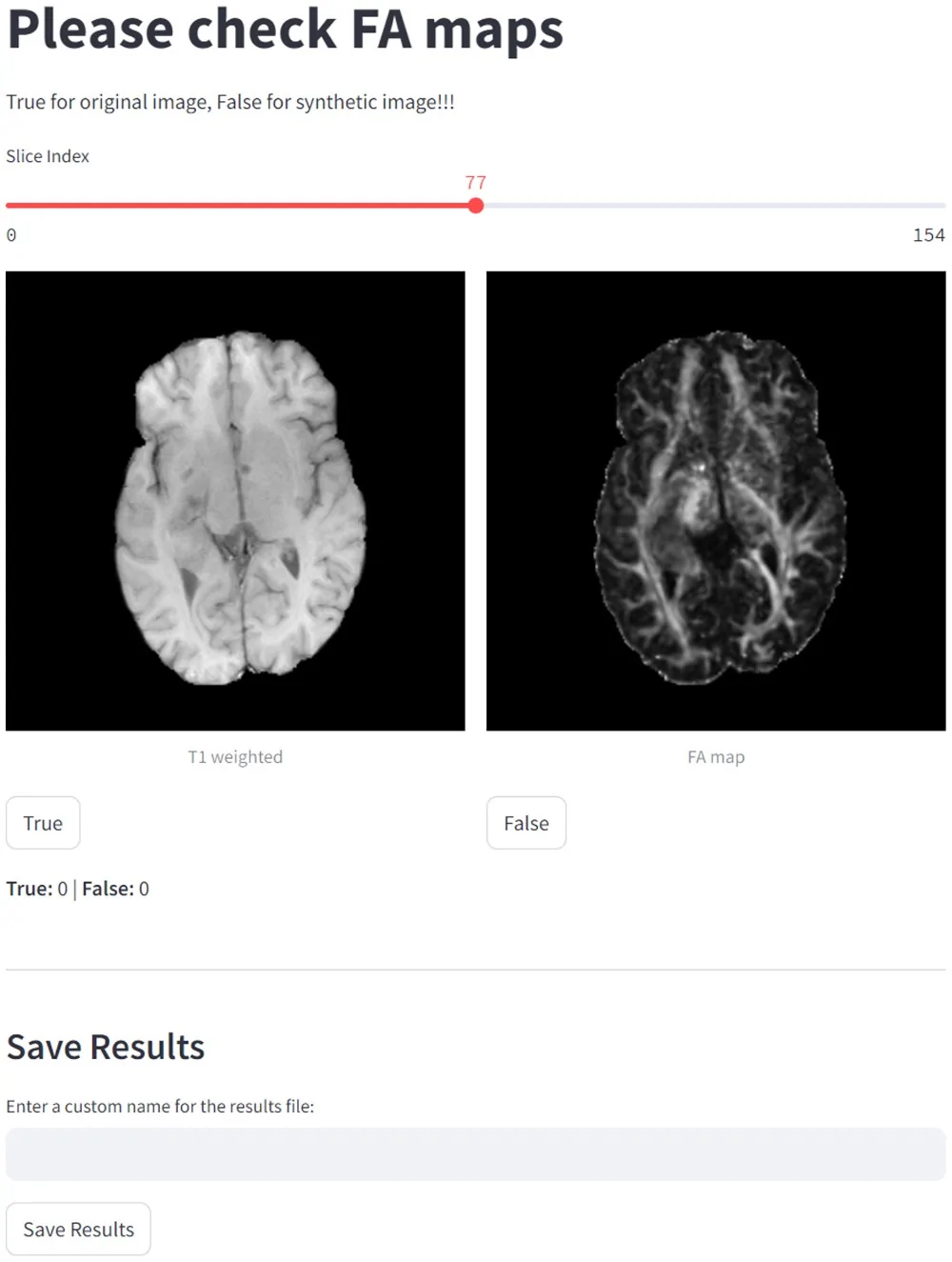
Screenshot of the application used to assess the quality of generated images by allowing radiologists to distinguish between synthetic and ground truth maps. The interface provides options to label images as “True” for ground truth and “False” for generated images. For each case, both T1W images and FA maps are displayed, with an interactive slider enabling the visualization of different slices from the 3D scan. The purpose of this is not to assess alignment between ground truth and synthetic maps but to evaluate the hallucination—or false features—present in the synthetic maps.

#### Structural similarity index

Structural similarity index is a perceptual metric that quantifies the similarity between 2 images by considering luminance, contrast, and structural information.^47^ Unlike traditional pixel-wise metrics, SSIM accounts for human visual perception, making it more robust to variations in intensity and contrast. It is computed as:


(5) $$\mathrm{SSIM}(x,y)=\frac{\left(2\mu_{x}\mu_{y}+C_{1})(2\sigma_{xy}+C_{2}\right)}{\left(\mu_{x}^{2}+\mu_{y}^{2}+C_{1})(\sigma_{x}^{2}+\sigma_{y}^{2}+C_{2}\right)}$$

where $\mu_{x}$ and $\mu_{y}$ are the mean intensities of images *x* and *y*, $\sigma_{x}^{2}$ and $\sigma_{y}^{2}$ are their variances, and $\sigma_{xy}$ represents their covariance. Constants $C_{1}$ and $C_{2}$ stabilize the division. SSIM values range from −1 to 1, where 1 indicates perfect similarity.

#### Multi-scale structural similarity index

Multi-scale structural similarity index extends SSIM by evaluating image similarity at multiple scales, enhancing its ability to capture structural distortions across different resolutions.^48^ Instead of computing SSIM at a single scale, MS-SSIM applies a hierarchical approach by downsampling the image iteratively and computing SSIM at each level. The overall MS-SSIM score is computed as a weighted product of SSIM values across scales:


(6) $$\text{MS-SSIM}(x,y)=\prod_{i=1}^{M}{\left[{\text{SSIM}}_{i}(x,y)\right]}^{\alpha_{i}}$$

where ${\text{SSIM}}_{i}$ represents the SSIM value at scale *i*, and $\alpha_{i}$ are weighting factors. MS-SSIM provides improved perceptual correlation compared to SSIM, particularly for images with varying spatial frequencies.

### Peak signal-to-noise ratio

Peak signal-to-noise ratio is a commonly used metric that measures the ratio between the maximum possible signal power and the power of corrupting noise in an image.^49^ It is defined as:


(7) $$\text{PSNR}=10{\;\text{log}\;}_{10}\left(\frac{{\text{MAX}}_{I}^{2}}{\text{MSE}}\right)$$

where ${\text{MAX}}_{I}$ is the maximum pixel intensity value (eg, 255 for 8-bit images), and MSE is the mean squared error between the generated and ground truth images. Higher PSNR values indicate better image quality, with typical values ranging from 20 to 50 dB, depending on image fidelity.

## Experiments

This study conducted 4 distinct experiments to evaluate the proposed framework from both quantitative and qualitative perspectives. The first experiment involved training a model exclusively on healthy brain data to assess its ability to generate synthetic FA maps. The second experiment focused on training a separate model on tumor-affected brain data to understand its performance in pathological contexts. The third experiment explored transfer learning by adapting the model trained on healthy brains to tumor cases, examining its generalization capabilities. Finally, the fourth experiment assessed the fidelity of the synthetic DEC maps in reflecting the underlying orientational information of white matter for the healthy cases.

### Synthetic maps for healthy cases

Figure 3 presents the generated FA and DEC maps across 3 anatomical planes. The synthetic maps closely resemble the ground truth, particularly in major white matter tracts such as the corpus callosum and corticospinal tract. Although the synthetic maps exhibit slightly lower resolution compared to the ground truth, the white matter trajectories remain well-defined, making them suitable for subsequent attention region connection analysis in the clinical pipeline.

**Figure 3:**
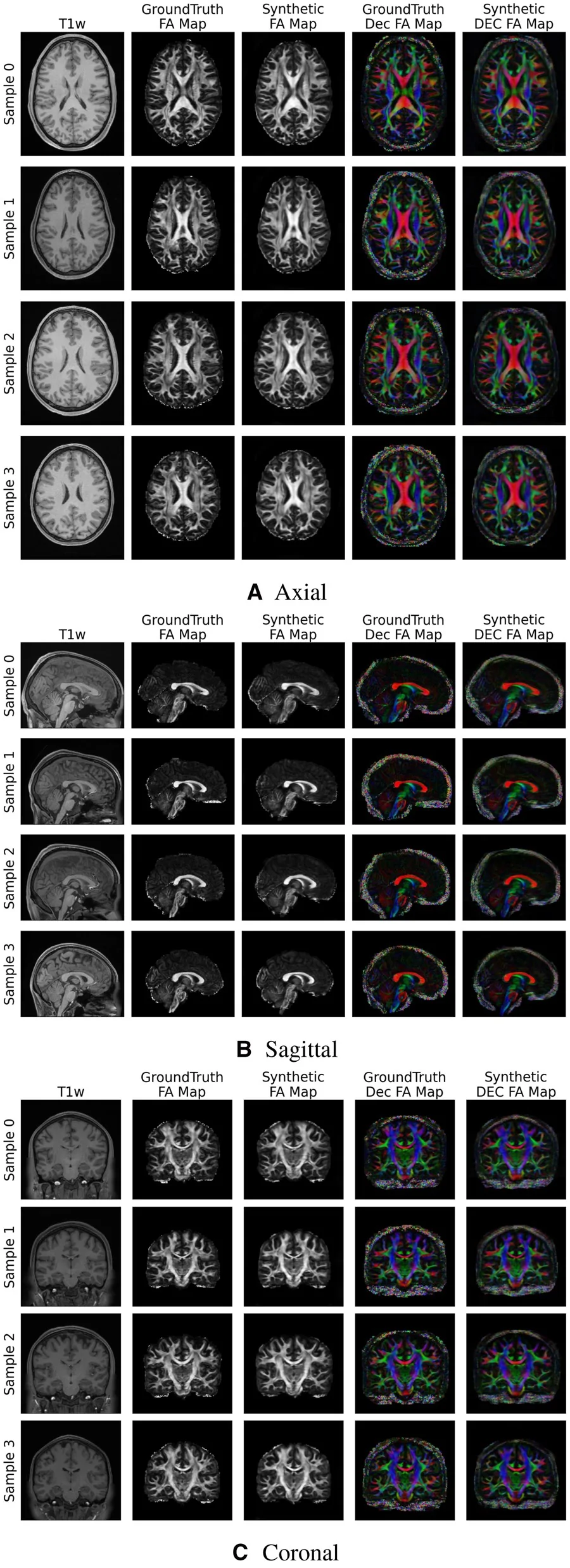
Synthetic 3D FA and DEC maps of healthy brains shown in (A) axial, (B) sagittal, and (C) coronal views. The first column shows T1W scans as input, the second and fourth columns present ground truth FA and DEC maps, and the third and fifth columns display the corresponding synthetic maps.

For short-range white matter connections, most structures are accurately generated with a strong correspondence to the ground truth. However, some local connections exhibit minor morphological variations, and certain complex local connections in the synthetic DEC maps are not fully reconstructed. These limitations may stem from several factors. The finite receptive field of the convolutional kernels restricts the model’s ability to capture fine-grained local structural details, particularly in regions where white matter fibers undergo rapid directional changes or form densely packed bundles. In addition, the coarse, and directionally invariant nature of white matter signal visualized in T1WI limits the ability to recapture fine grained microstructure, especially for short-range connections. Furthermore, while the cycle consistency loss enforces global structural, it does not impose explicit constraints on local fiber directionality, which may limit the model’s capacity to faithfully reconstruct subtle directional transitions in complex white matter regions. Future work could explore strategies such as dilated convolutions, larger kernel sizes, or attention mechanisms to enlarge the effective receptive field, as well as incorporating direction-aware loss terms to better supervise the synthesis of complex local white matter architecture.

### Synthetic maps for cases with tumor in situ

Figure 4 presents a comparison between learning from scratch and transfer learning on tumor cases as the percentage of training data increases from 1% to 100% (approximately 5-500 samples). When utilizing the full set of training samples, both methods effectively capture the tumor regions in the generated maps. Furthermore, the long-range white matter connections between normal regions are accurately and clearly represented. However, the fidelity of the generated maps is visually lower than the ground truth, exhibiting partially obscured areas, a loss of fine details, and imprecise boundaries.

**Figure 4:**
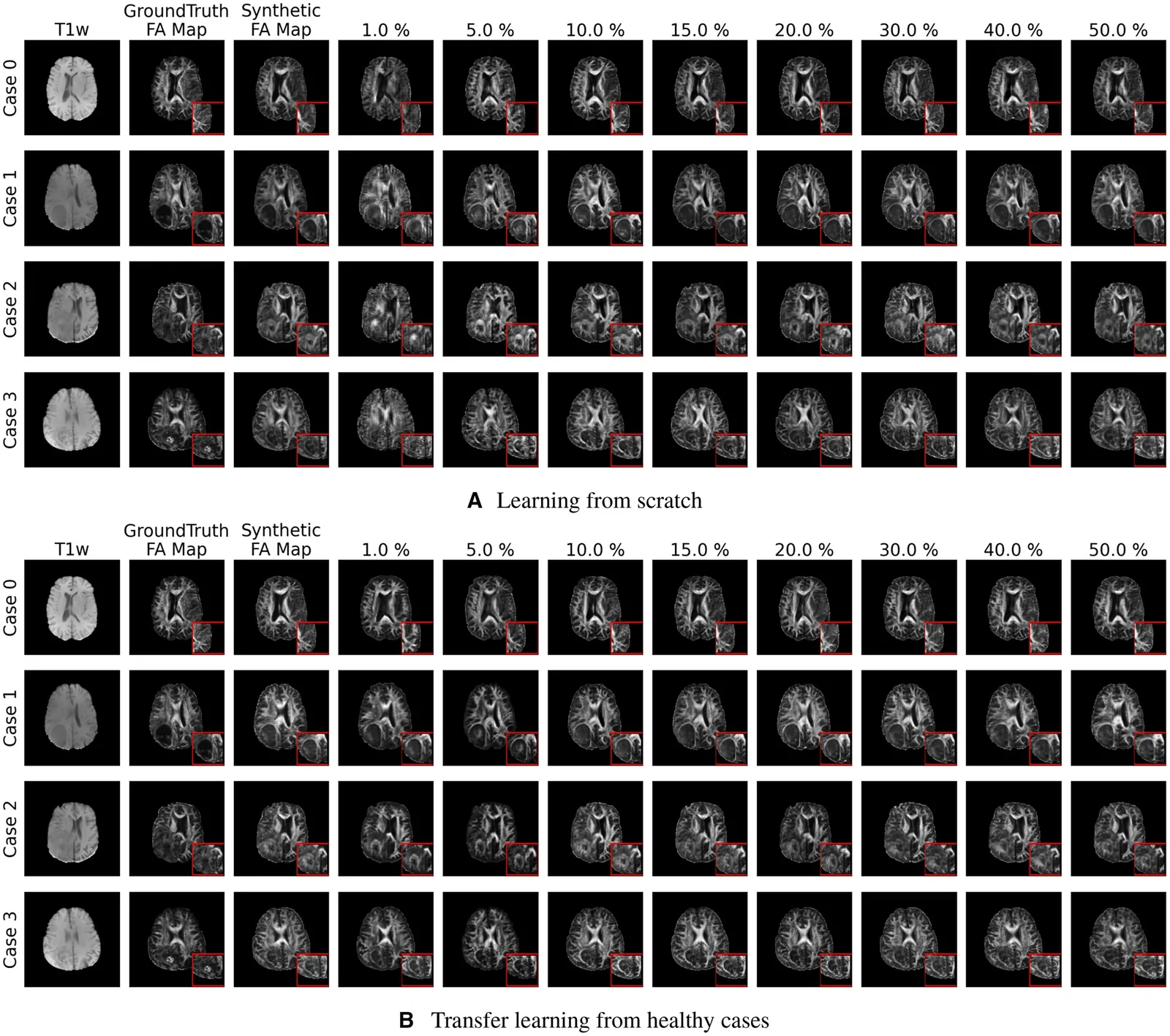
Synthetic FA maps of the brain with tumors generated using (A) learning from scratch and (B) transfer learning. The percentages indicate the proportion of training samples used for model training.

When only a limited number of samples (1%) are available for training the model, transfer learning demonstrates significantly better performance compared to learning from scratch with the same dataset. This improvement is primarily reflected in a clearer representation of structural features and a more explicit delineation of tumor regions. Nevertheless, due to the limited training data, the generated tumor regions from transfer learning still exhibit noticeable discrepancies compared to the ground truth. As additional training data is introduced, the difference between transfer learning and learning from scratch progressively decreases (approximately up to 30%).

### Quantitative analysis

Figure 5 compares the generated FA maps with the ground truth at the voxel-wise level. The average intensity of each scan is computed for both the generated and ground truth maps for each case. The results show a distinct distribution between tumor and healthy cases. Specifically, the mean intensity value for each 3D scan in healthy cases is approximately 0.055, whereas tumor cases exhibit lower values.

**Figure 5:**
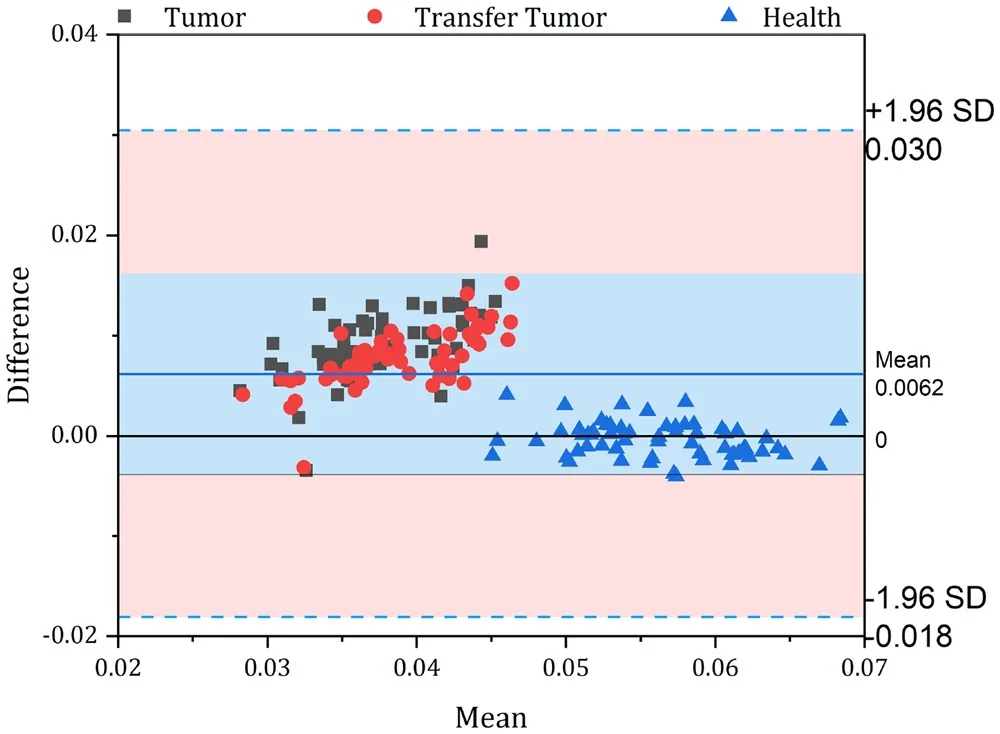
Bland-Altman plot comparing the generated FA maps with the ground truth for both healthy and tumor cases (encompassing results of learning from scratch and transfer learning approaches).

Furthermore, the difference between the model-generated and ground truth maps is statistically lower in healthy cases compared to tumor cases, indicating that the current model performs better in generating FA maps for healthy brains. Additionally, the performance of tumor case generation using transfer learning shows a slightly lower difference compared to training from scratch, suggesting that leveraging knowledge from healthy cases improves the generation maps in tumor-affected cases.

Table 1 and Figure 6 present the evaluation of generation quality using SSIM, MS-SSIM, and PSNR. Consistent with the visual assessment, the generated scans for healthy cases demonstrate better performance compared to tumor cases. This discrepancy may be attributed to the higher input quality and a larger number of training samples available for healthy cases.

**Figure 6:**
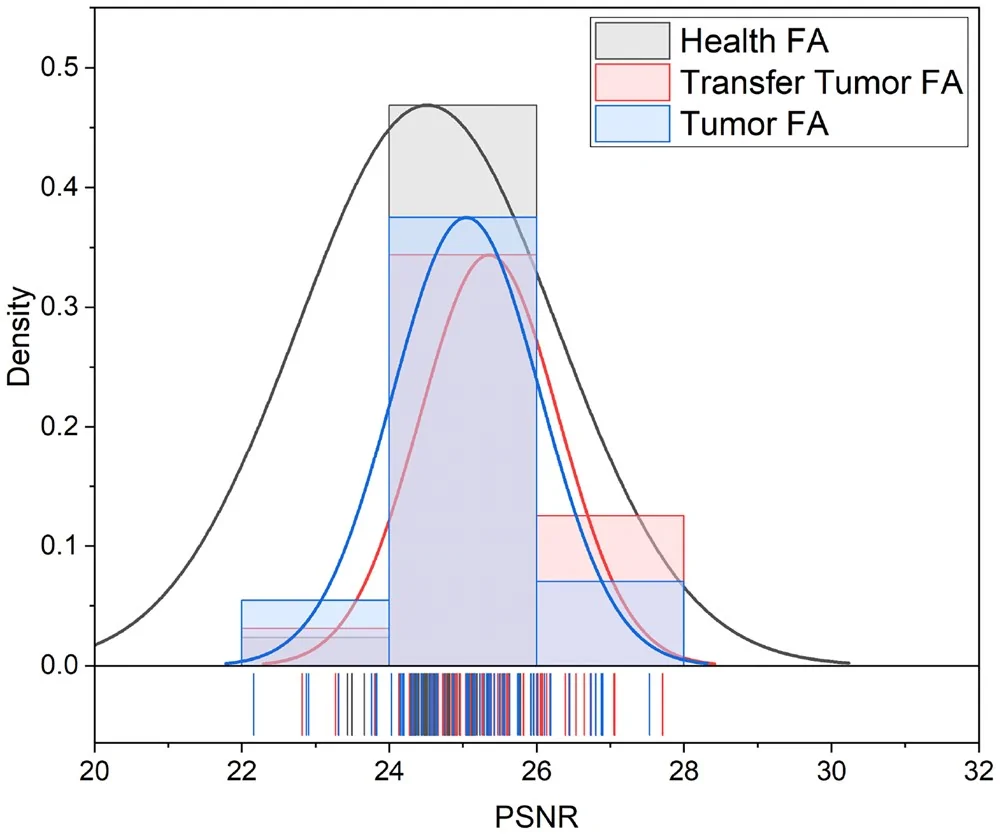
Quantitative evaluation of the generated FA maps using PSNR. Higher PSNR values indicate lower reconstruction error and better image quality.

**Table 1:** Quantitative evaluation of the generated images using SSIM and MS-SSIM.

Metric	SSIM	MS-SSIM
**Healthy FA**	0.879	0.975
**Tumor FA**	0.883	0.958
**Transfer tumor FA**	0.886	0.964
**Healthy DEC**	0.700	0.932

Figure 7 evaluates the spatial agreement between binarized real and synthesized FA maps within the tumor ROI through a threshold sensitivity analysis. The curve shows a clear monotonic decrease in mean Dice score as the binarization threshold increases from 0.05 to 0.95, with peak performance at 0.05 (mean Dice = 0.9724±0.025), indicating high spatial agreement when nearly all non-zero tissue is included. This behavior is expected given the pathophysiological characteristics of tumor tissue: FA values within the tumor ROI are inherently low due to disrupted white matter architecture and increased cellularity, meaning the majority of voxels are concentrated in the lower intensity range. As the threshold rises, progressively fewer voxels survive binarization in both the real and synthesized maps, and Dice becomes increasingly sensitive to minor spatial discrepancies between the 2 sparse masks, driving the steep decline observed beyond 0.10 and the drop below 0.50 beyond 0.25. The widening SD in the mid-threshold range (0.10-0.30) reflects inter-case variability in the extent to which residual organized white matter tracts traverse or border the tumor, rather than systematic failure of the generator. Overall, a threshold of 0.05 is reasonable for evaluating synthesized FA structural fidelity within the tumor ROI, confirming that the generator reliably reproduces the spatial extent of tumor tissue occupancy, though the rapid Dice decline at higher thresholds suggests that precise localization of residual high-FA structures within the tumor remains challenging.

**Figure 7:**
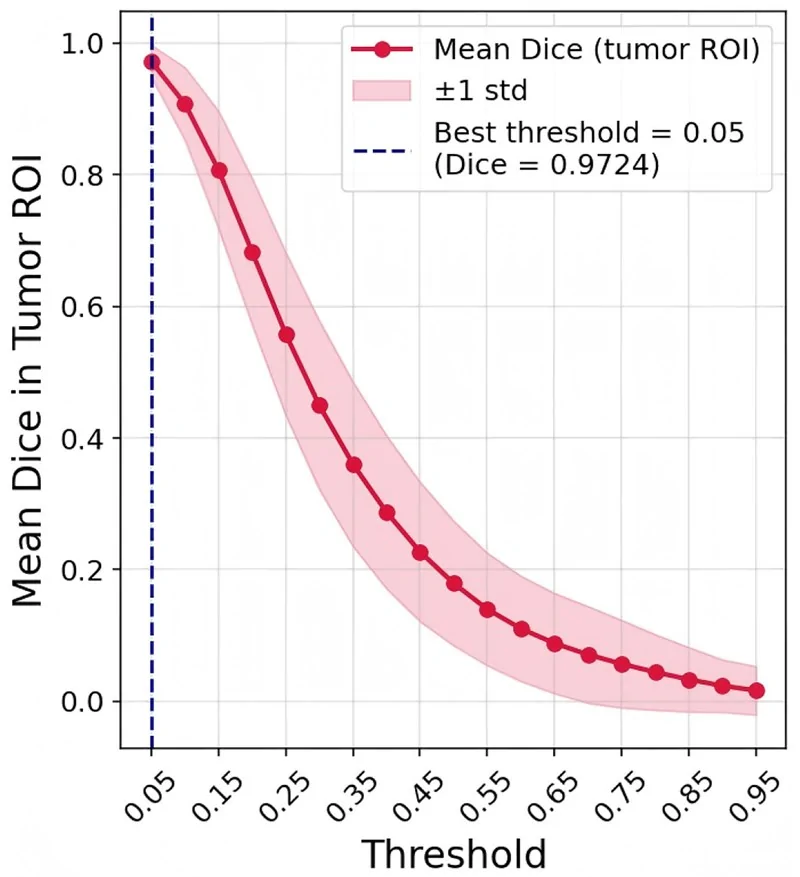
Threshold sensitivity analysis for binarized FA map comparison within the tumor ROI. The mean DSC between binarized real and synthesis FA maps is plotted as a function of the binarization threshold, evaluated exclusively within the tumor ROI defined by the ground-truth mask. The shaded band represents ±1 SD across cases. The optimal threshold is indicated by the dashed vertical line.

For the DEC map, the generation performance is notably lower than that of FA maps, indicating greater challenges in accurately synthesizing DEC images. Furthermore, leveraging knowledge transfer enhances the quality of generated tumor case maps, as reflected by an increase in the number of cases with higher PSNR values compared to training without transfer learning.

To further analyze the effects of transfer learning, Figure 8 compares the generated images obtained with and without transfer learning. Additionally, it examines the differences between normal tissues (where the tumor is masked) and tumor tissues to assess the preservation of anatomical structures.

**Figure 8:**
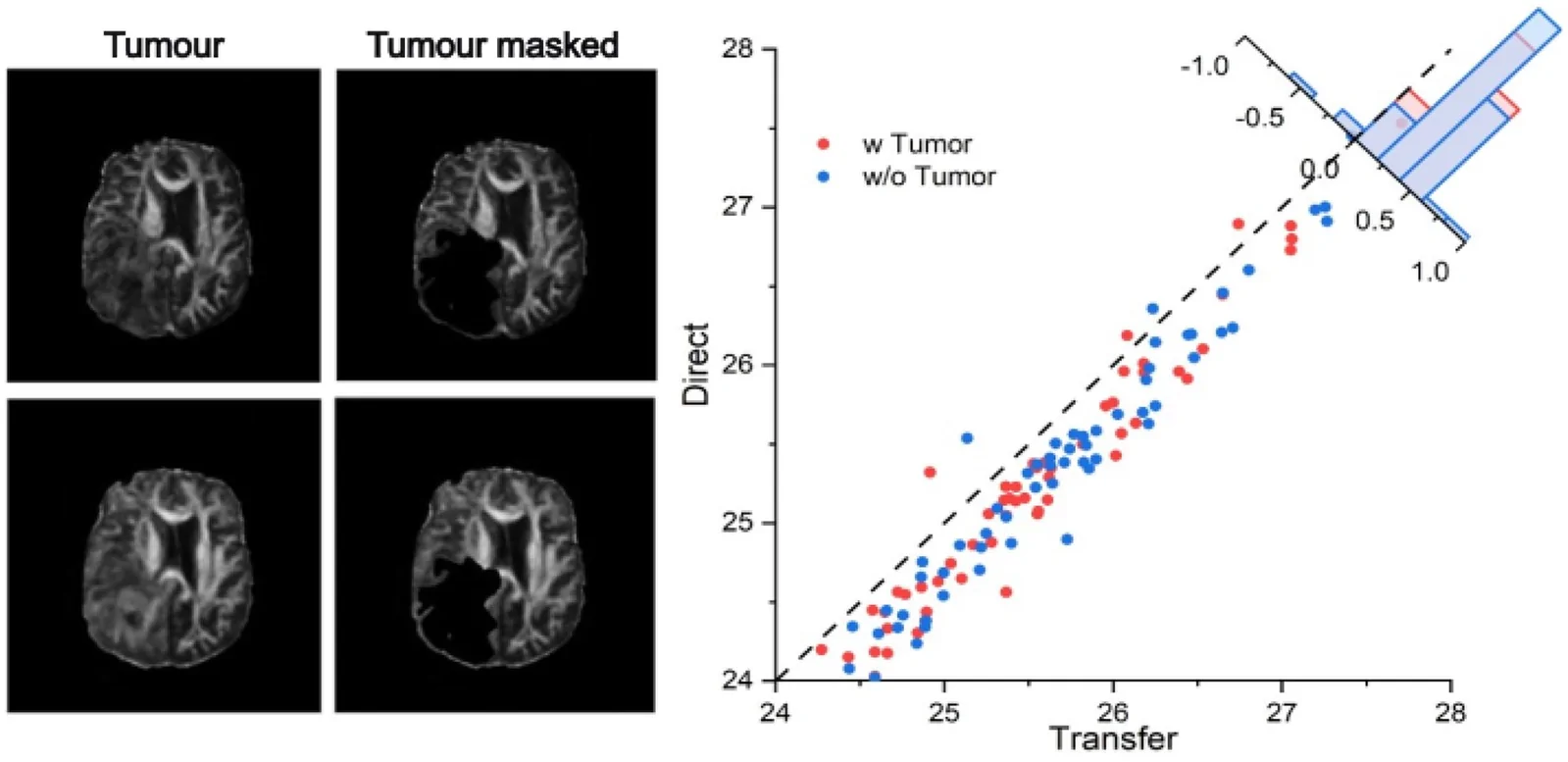
Comparison of generated images with and without tumor masking based on PSNR evaluation. The left subfigure provides an illustration of the tumor masking conditions. The right subfigure presents the PSNR distribution for cases where tumor masking is applied or not, under both transfer learning and direct learning settings. Additionally, the bar plot in the top right corner visualizes the distribution of case-wise PSNR differences between transfer learning and direct learning (transfer-direct), providing insights into the impact of transfer learning on reconstruction quality.

The results indicate that for both the whole brain and the brain regions excluding the tumor, transfer learning slightly but consistently outperforms direct learning in terms of PSNR. As shown in the scatter plot, the majority of data points fall below the diagonal dashed line, indicating that transfer learning yields higher reconstruction quality across most cases. This improvement is observed in both tumor-affected regions and normal tissue areas, suggesting that the performance gain is not confined to pathological structures but extends to the broader anatomical context.

These findings carry important implications for the role of prior knowledge in medical image synthesis. By initializing the model with weights learned from healthy brain data, transfer learning enables the network to leverage established representations of normal anatomical structures. This preexisting knowledge appears to act as a form of structural regularization, helping the model maintain overall anatomical consistency even when adapting to pathological cases. In particular, the preservation of reconstruction quality in tumor-masked regions suggests that healthy-brain knowledge contributes to the faithful synthesis of surrounding normal tissue, which is critical for downstream clinical tasks such as tumor delineation and radiological assessment. The bar plot in the top right corner further corroborates this, showing that the distribution of case-wise PSNR differences (transfer − direct) is positively skewed, with transfer learning yielding gains across the majority of cases.

Taken together, these results support the hypothesis that knowledge transferred from healthy brains not only improves tumor region synthesis but also reinforces global anatomical plausibility, underscoring the value of transfer learning as a strategy for robust and clinically meaningful MRI synthesis.

Figure 9 provides a comparison of different evaluation metrics to track the correlation between PSNR and either SSIM or MS-SSIM for the tumor region alone, the brain with the tumor masked, and the whole brain. Notably, PSNR shows a stronger positive correlation with SSIM than with MS-SSIM. Additionally, all evaluation metrics demonstrate significantly higher performance on the tumor region alone compared to the brain with the tumor masked or the whole brain. This indicates that the model is better at capturing the details of the tumor during the generation process.

**Figure 9:**
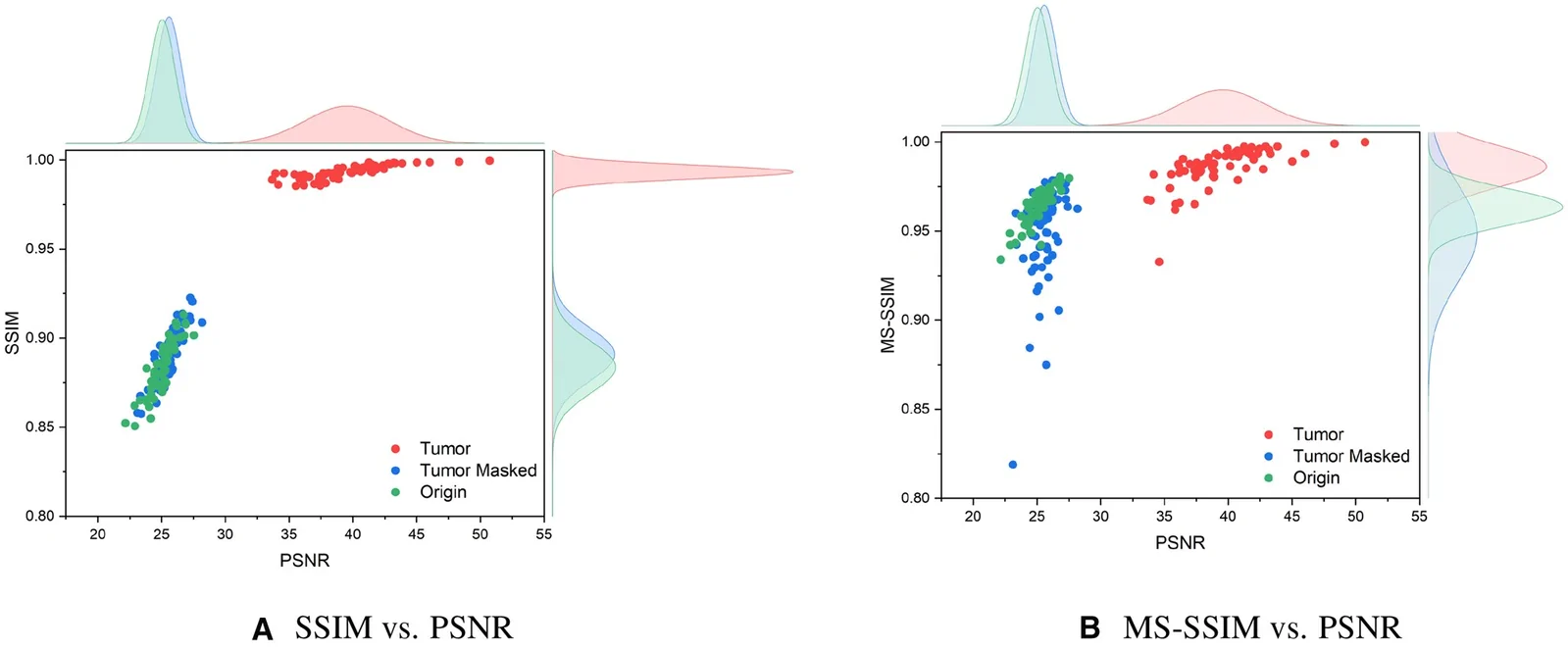
Comparison of generated image quality between the tumor region, the region after tumor masking, and the whole brain image. (A) SSIM versus PSNR. (B) MS-SSIM versus PSNR. Each point represents one subject, and the marginal curves show the distribution of each metric. The evaluation tracks the differences in image quality across these distinct areas.

### Radiologist check

Additionally, 2 experienced radiologists were invited to review the generated maps and determine whether they were synthetic or ground truth, using a developed app. The results showed that 1 radiologist achieved 60% accuracy, while the other achieved 50% accuracy after reviewing 60 scans. These findings suggest that, from a clinical perspective, the quality of the generated maps is satisfactory, as the radiologists were not able to correctly identify the real maps with a reasonably high degree of accuracy.

## Discussion

In this study, we have used several experiments to demonstrate the feasibility of using a CycleGAN framework for generating FA and DEC maps. We have evaluated 4 different scenarios, 1 of which involves scans with the presence of a brain tumor that disturbs normal underlying architecture; using 3 distinct evaluation metrics, we rendered clinically useful imaging maps created in the exact same reference frame as the T1WI that were used to generate them. The accuracy of the generated maps is sufficient to improve spatial misalignment among structural and diffusion-derived images, such as FA.

Despite the utility of DEC and FA as previously discussed, they are not ubiquitously used maps, because the acquisition of DTI is not standardized or routine in the clinical setting.^50^ This is due to both intrinsic and extrinsic limitations, which include imaging artifacts, lack of standardized techniques, as well as variations in the preprocessing and post-processing of the imaging data.^50^ Additionally, as DTI accuracy increases with the number of encoding directions, more encoding directions equates lengthier scan time, which may not be conducive for patients.^51^^,^^52^ Moreover, DTI incurs additional costs and resources, with a considerable added expense stemming from post-processing of collected DTI data.^53^

Nonetheless, these limitations do not negate the clinical utility of DTI-derived scalar metrics and images, which have practical implications for a neurosurgeon’s preoperative planning. Fortunately, our CycleGAN model addresses and mitigates some of the DTI processing, patient-centered, and cost-associated limitations. The ability to generate DTI-derived maps, such as FA and DEC, from conventional T1-weighted MR images, without the need for additional DTI acquisition steps, circumvents several technical limitations. Also, the resource and cost burdens of capturing DTI are similarly avoided, removing the additional step of post-processing these images using proprietary software. Even more, the need to extend the scan time for patients who may already be averse to the duration for conventional MRI acquisition is obviated.

The synthetic generation of high-fidelity FA and DEC maps affords physicians, particularly neurosurgical oncologists, the practical and clinical benefit that these images can offer, overcoming several obstacles that are otherwise encountered when collecting DTI data. DTI is not a routine or ubiquitous imaging modality, but using this technology, neurosurgeons can readily generate FA and DEC images from T1WI scans that are already routinely obtained, offering both the surgeon and the patient unhindered access to the information that these images provide. The surgeon can use them for preoperative planning and as a visual aid when explaining the course of action to patients with brain tumors contemplating neurosurgery.

This would be particularly useful for patients with an aggressive brain tumor like glioblastoma, which accounts for 48.6% of all malignant tumors of the central nervous system, and is treated with maximal safe surgical resection as part of a standard of care.^54–57^ While many patients may not have access to centers that offer DTI, this boundary to access would no longer be a limitation to surgeons and patients who could otherwise benefit from what DTI visualization of brain connectivity has to offer. Furthermore, while there remains room for refinement of this technology, we demonstrate the feasibility of synthetically generating FA and DEC maps while maintaining the overall structural integrity of predominant white matter fiber pathways. In all, this may lend a step toward more routine and resource-efficient use of these valuable, noninvasive imaging assets for surgical and radiotherapy treatment planning in brain tumor patients.

While diffusion models have demonstrated impressive image synthesis quality in recent studies, they typically require significantly higher computational resources and longer inference times, which may limit their practicality in clinical workflows. In pairwise settings, they can also be sensitive to registration errors, as misalignment between paired images may degrade supervision quality and compromise synthesis performance. Moreover, their application to 3D volumetric medical data remains computationally challenging and is still an active area of research. Our CycleGAN framework is well-suited for unpaired image-to-image translation, which is particularly advantageous in the medical imaging context where paired multi-modal data are difficult to obtain. Regarding tumor-affected brains specifically, standard GAN-based methods trained predominantly on healthy subjects often struggle to preserve pathological features during synthesis, as tumor regions introduce irregular intensity distributions and structural anomalies that deviate significantly from normal anatomy. Our approach addresses this challenge through the incorporation of the Cor-Coe Loss and transfer learning from healthy brain data, enabling the model to maintain anatomical consistency while faithfully representing tumor-associated features in the synthetic FA maps.

Data privacy and security are critical considerations when handling sensitive medical data such as MRI scans and tumor pathology. In this study, all data were anonymized and processed in compliance with relevant ethical and data protection regulations. Furthermore, the proposed model operates solely on de-identified imaging data, minimizing privacy risks. For future clinical deployment, additional measures such as secure data governance, access control, and privacy-preserving learning frameworks (eg, federated learning) will be essential to ensure compliance with healthcare data standards.

## Conclusion

In this study, we investigated the feasibility of generating DTI scans, including FA and DEC maps, for both healthy and tumor-affected brains using Cycle-GAN models. The quality of the generated maps was assessed through quantitative and qualitative metrics as well as expert evaluation by radiologists. The results demonstrate that the synthetic maps effectively capture long-range white matter connections, preserving the overall structural integrity of major white matter tracts. However, minor deformations are observed in the representation of local white matter connections, particularly in regions with complex arrangements. These findings suggest that while the proposed approach holds promise for generating high-fidelity DTI data, further refinements are needed to enhance the accuracy of local structural details. Future work will focus on improving the resolution and anatomical precision of the generated maps, ensuring their robustness for clinical and research applications.

## Author contributions

Xin Du: Conceptualization, Methodology, Software, Formal analysis, Validation, Investigation, Writing – original draft, Writing – review & editing. Francesca M. Cozzi: Writing – review & editing. Stephen J. Price: Resources, Supervision, Writing – review & editing. Rajesh Jena: Supervision, Funding acquisition, Writing – review & editing.

## Data Availability

The data underlying this article are available from third-party public repositories. Healthy-cohort data were provided by the MGH-USC Human Connectome Project (HCP; U01-MH93765; Principal Investigators: Bruce Rosen, MD, PhD, Arthur W. Toga, PhD, and Van J. Wedeen, MD), funded by the National Institute of Dental and Craniofacial Research, the National Institute of Mental Health, and the National Institute of Neurological Disorders and Stroke. These data are disseminated by the Laboratory of Neuro Imaging at the University of Southern California and are available at https://ida.loni.usc.edu, subject to the HCP data use terms. Tumour-cohort data were obtained from the University of California San Francisco Preoperative Diffuse Glioma MRI (UCSF-PDGM) dataset, available from The Cancer Imaging Archive (TCIA) at https://doi.org/10.7937/tcia.bdgf-8v37 under the TCIA data usage policy. No new data were generated in this study.

## References

[1] Jones DK. Gaussian modeling of the diffusion signal. In: Diffusion MRI: From Quantitative Measurement to in-Vivo Neuroanatomy. 2nd ed. San Diego, CA: Academic Press/ Elsevier; 2014:87-104.

[2] Tae W-S , Ham B-J, Pyun S-B, Kang S-H, Kim B-J. Current clinical applications of diffusion-tensor imaging in neurological disorders. J Clin Neurol (Seoul, Korea). 2018;14:129-140. 10.3988/jcn.2018.14.2.129 PMC5897194 29504292

[3] van den Elshout R , Ariëns B, Blaauboer J, et al Quantification of perineural satellitosis in pretreatment glioblastoma with structural MRI and a diffusion tensor imaging template. Neurooncol Adv. 2024;6:vdad168. 10.1093/noajnl/vdad168 PMC10776201 38196738

[4] Scherer H. Structural development in gliomas. Am J Cancer. 1938;34:333-351.

[5] Scherer H. The forms of growth in gliomas and their practical significance. Brain. 1940;63:1-35. 10.1093/brain/63.1.1

[6] Matsukado Y , MacCarty CS, Kernohan JW. The growth of glioblastoma multiforme (astrocytomas, grades 3 and 4) in neurosurgical practice. J Neurosurg. 1961;18:636-644. 10.3171/jns.1961.18.5.0636 13768222

[7] Drumm MR , Dixit KS, Grimm S, et al Extensive brainstem infiltration, not mass effect, is a common feature of end-stage cerebral glioblastomas. Neuro Oncol. 2020;22:470-479. 10.1093/neuonc/noz216 PMC7158646 31711239

[8] Latini F , Fahlström M, Beháňová A, et al The link between gliomas infiltration and white matter architecture investigated with electron microscopy and diffusion tensor imaging. Neuroimage Clin. 2021;31:102735. 10.1016/j.nicl.2021.102735 PMC8274339 34247117

[9] Schubert MC , Soyka SJ, Tamimi A, et al Deep intravital brain tumor imaging enabled by tailored three-photon microscopy and analysis. Nat Commun. 2024;15:7383. 10.1038/s41467-024-51432-4 PMC11387418 39256378

[10] Brooks LJ , Clements MP, Burden JJ, et al The white matter is a pro-differentiative niche for glioblastoma. Nat Commun. 2021;12:2184. 10.1038/s41467-021-22225-w PMC8042097 33846316

[11] Esmaeili M , Stensjøen AL, Berntsen EM, Solheim O, Reinertsen I. The direction of tumor growth in glioblastoma patients. Sci Rep. 2018;8:1199. 10.1038/s41598-018-19420-z PMC5775193 29352231

[12] Cozzi FM , Mayrand RC, Wan Y, Price SJ. Predicting glioblastoma progression using mr diffusion tensor imaging: a systematic review. J Neuroimaging. 2025;35:e13251. 10.1111/jon.13251 PMC11626419 39648937

[13] Metz M-C , Molina-Romero M, Lipkova J, et al Predicting glioblastoma recurrence from preoperative MR scans using fractional-anisotropy maps with free-water suppression. Cancers (Basel). 2020;12:728. 10.3390/cancers12030728 PMC7140058 32204544

[14] Jin Y , Randall JW, Elhalawani H, et al Detection of glioblastoma subclinical recurrence using serial diffusion tensor imaging. Cancers (Basel). 2020;12:568. 10.3390/cancers12030568 PMC7139975 32121471

[15] Schneider JR , Raval AB, Black K, Schulder M. Diffusion tensor imaging color-coded maps: an alternative to tractography. Stereotact Funct Neurosurg. 2021;99:295-304. 10.1159/000512092 33461209

[16] Witwer BP , Moftakhar R, Hasan KM, et al Diffusion-tensor imaging of white matter tracts in patients with cerebral neoplasm. J Neurosurg. 2002;97:568-575. 10.3171/jns.2002.97.3.0568 12296640

[17] Wang L-W , Cho K-H, Chao P-Y, et al White and gray matter integrity evaluated by MRI-DTI can serve as noninvasive and reliable indicators of structural and functional alterations in chronic neurotrauma. Sci Rep. 2024;14:7244. 10.1038/s41598-024-57706-7 PMC10973494 38538745

[18] Feng Y , Chandio BQ, Villalon-Reina JE, et al; Alzheimer’s Disease Neuroimaging Initiative. Microstructural mapping of neural pathways in Alzheimer’s disease using macrostructure-informed normative tractometry, Alzheimer’s Dement. 2025;21:e14371. 10.1002/alz.14371 PMC11782200 39737627

[19] Basser PJ , Pajevic S, Pierpaoli C, Duda J, Aldroubi A. In vivo fiber tractography using DT-MRI data. Magn Reson Med. 2000;44:625-632. 10.1002/1522-2594(200010)44:4<625::AID-MRM17>3.0.CO; 2-O 11025519

[20] Le Bihan D , Iima M. Diffusion magnetic resonance imaging: what water tells us about biological tissues. PLoS Biol. 2015;13:e1002203. 10.1371/journal.pbio.1002203 PMC4559450 26334873

[21] Van Essen DC , Dierker DL. Surface-based and probabilistic atlases of primate cerebral cortex. Neuron. 2007;56:209-225. 10.1016/j.neuron.2007.10.015 17964241

[22] Jones DK , Knösche TR, Turner R. White matter integrity, fiber count, and other fallacies: the do’s and don’ts of diffusion MRI. Neuroimage. 2013;73:239-254. 10.1016/j.neuroimage.2012.06.081 22846632

[23] Karimi D , Warfield SK. Diffusion MRI with machine learning. Imaging Neuroscience. 2024;2:1-55. 10.1162/imag_a_00353 PMC11981007 40206511

[24] Preetha CJ , Meredig H, Brugnara G, et al Deep-learning-based synthesis of post-contrast t1-weighted MRI for tumor response assessment in neuro-oncology: a multicentre, retrospective cohort study. Lancet Digit Health. 2021;3:e784-e794. 10.1016/S2589-7500(21)00205-3 34688602

[25] Herrmann J , Benkert T, Brendlin A, et al Shortening acquisition time and improving image quality for pelvic MRI using deep learning reconstruction for diffusion-weighted imaging at 1.5 t. Acad Radiol. 2024;31:921-928. 10.1016/j.acra.2023.06.035 37500416

[26] Abu-Srhan A , Almallahi I, Abushariah MA, Mahafza W, Al-Kadi OS. Paired-unpaired unsupervised attention guided gan with transfer learning for bidirectional brain MR-CT synthesis. Comput Biol Med. 2021;136:104763. 10.1016/j.compbiomed.2021.104763 34449305

[27] Zhu J-Y , Park T, Isola P, Efros AA. Unpaired image-to-image translation using cycle-consistent adversarial networks. In: *Proceedings of the IEEE International Conference on Computer Vision,* October 22-29, 2017, Venice, Italy. IEEE; 2017:2223-2232.

[28] Dayarathna S , Islam KT, Uribe S, Yang G, Hayat M, Chen Z. Deep learning based synthesis of MRI, CT and PET: review and analysis. Med Image Anal. 2024;92:103046. 10.1016/j.media.2023.103046 38052145

[29] Hu Y , Zhou H, Cao N, Li C, Hu C. Synthetic CT generation based on cbct using improved vision transformer cyclegan. Sci Rep. 2024;14:11455. 10.1038/s41598-024-61492-7 PMC11106312 38769329

[30] Diniz E , Santini T, Karim H, Aizenstein HJ, Ibrahim TS. Cross-Modality image translation of 3 Tesla Magnetic Resonance Imaging to 7 Tesla using generative adversarial networks. Hum Brain Mapp. 2025;46:e70246. 10.1002/hbm.70246 PMC12182983 40545512

[31] Farshchitabrizi AH , Sadeghi MH, Sina S, Alavi M, Feshani ZN, Omidi H. AI-enhanced PET/CT image synthesis using CycleGAN for improved ovarian cancer imaging. Pol J Radiol. 2025;90:e26-e35. 10.5114/pjr/196804 PMC11891552 40070416

[32] Gong C , Huang Y, Luo M, et al Channel-wise attention enhanced and structural similarity constrained cyclegan for effective synthetic CT generation from head and neck MRI images. Radiat Oncol. 2024;19:37. 10.1186/s13014-024-02429-2 PMC10938692 38486193

[33] Wolterink JM , Dinkla AM, Savenije MH, Seevinck PR, van den Berg CA, Išgum I. Deep MR to CT synthesis using unpaired data. In: Simulation and Synthesis in Medical Imaging: Second International Workshop, SASHIMI 2017, Held in Conjunction with MICCAI 2017, Québec City, QC, Canada, September 10, 2017, Proceedings 2, Springer; 2017:14-23.

[34] Wang J , Wu QJ, Pourpanah F. Dc-cyclegan: bidirectional CT-to-MR synthesis from unpaired data. Comput Med Imaging Graph. 2023;108:102249. 10.1016/j.compmedimag.2023.102249 37290374

[35] Gu X , Knutsson H, Nilsson M, Eklund A. Generating diffusion MRI scalar maps from t1 weighted images using generative adversarial networks. In: Image Analysis: 21st Scandinavian Conference, SCIA 2019, Norrköping, Sweden: Springer; 2019:489-498.

[36] Huang X , Belongie S. Arbitrary style transfer in real-time with adaptive instance normalization. In: *Proceedings of the IEEE International Conference on Computer Vision*. Venice, Italy: IEEE; 2017:1501–1510.

[37] Cao S , Konz N, Duncan J, Mazurowski MA. Deep learning for breast MRI style transfer with limited training data. J Digit Imaging. 2023;36:666-678. 10.1007/s10278-022-00755-z PMC10039216 36544066

[38] Kazerouni A , Aghdam EK, Heidari M, et al Diffusion models in medical imaging: a comprehensive survey. Med Image Anal. 2023;88:102846. 10.1016/j.media.2023.102846 37295311

[39] Dhariwal P , Nichol A. Diffusion models beat GANS on image synthesis. Adv Neural Inf Process Syst. 2021;34:8780-8794.

[40] Kim J , Park H. Adaptive latent diffusion model for 3d medical image to image translation: Multi-modal magnetic resonance imaging study. In: *Proceedings of the IEEE/CVF Winter Conference on Applications of Computer Vision*. Waikoloa, HI, USA: IEEE; 2024:7604-7613.

[41] Glasser MF , Sotiropoulos SN, Wilson JA, et al; WU-Minn HCP Consortium. The minimal preprocessing pipelines for the human connectome project. Neuroimage. 2013;80:105-124. 10.1016/j.neuroimage.2013.04.127 PMC3720813 23668970

[42] Van Essen DC , Smith SM, Barch DM, et al; WU-Minn HCP Consortium. The wu-minn human connectome project: an overview. Neuroimage. 2013;80:62-79. 10.1016/j.neuroimage.2013.05.041 PMC3724347 23684880

[43] Calabrese E , Villanueva-Meyer JE, Rudie JD, et al The university of California San Francisco preoperative diffuse glioma MRI dataset. Radiol Artif Intell. 2022;4:e220058. 10.1148/ryai.220058 PMC9748624 36523646

[44] Ge Y , Wei D, Xue Z, et al Unpaired MR to CT synthesis with explicit structural constrained adversarial learning. In: 2019 IEEE 16th International Symposium on Biomedical Imaging (ISBI 2019). IEEE; 2019:1096-1099.

[45] Ge Y , Xue Z, Cao T, Liao S. Unpaired whole-body MR to CT synthesis with correlation coefficient constrained adversarial learning. In: Medical Imaging 2019: Image Processing. Vol. 10949. SPIE; 2019:28-35.

[46] Johnson J , Alahi A, Fei-Fei L. Perceptual losses for real-time style transfer and super-resolution. In: *Computer Vision-ECCV 2016: 14th European Conference*, Amsterdam, The Netherlands, October 11-14, 2016, Proceedings, Part II 14, Springer; 2016: 694-711.

[47] Wang Z , Bovik AC, Sheikh HR, Simoncelli EP. Image quality assessment: from error visibility to structural similarity. IEEE Trans Image Process. 2004;13:600-612. 10.1109/tip.2003.819861 15376593

[48] Wang Z , Simoncelli EP, Bovik AC. Multiscale structural similarity for image quality assessment. In: *The Thrity-Seventh Asilomar Conference on Signals, Systems & Computers*. Vol. 2. IEEE; 2003:1398-1402.

[49] Hore A , Ziou D. Image quality metrics: Psnr vs. ssim. In: 2010 20th International Conference on Pattern Recognition. IEEE; 2010: 2366-2369.

[50] Manan AA , Yahya N, Idris Z, Manan HA. The utilization of diffusion tensor imaging as an image-guided tool in brain tumor resection surgery: a systematic review. Cancers (Basel). 2022;14:2466. 10.3390/cancers14102466 PMC9139820 35626069

[51] Kumpulainen V , Merisaari H, Copeland A, et al Effect of number of diffusion-encoding directions in diffusion metrics of 5-year-olds using tract-based spatial statistical analysis. Eur J Neurosci. 2022;56:4843-4868. 10.1111/ejn.15785 PMC9545012 35904522

[52] Alexander AL , Lee JE, Lazar M, Field AS. Diffusion tensor imaging of the brain. Neurotherapeutics. 2007;4:316-329. 10.1016/j.nurt.2007.05.011 PMC2041910 17599699

[53] Hancock C , Bernal B, Medina C, Medina S. Cost analysis of diffusion tensor imaging and MR tractography of the brain. OJRad. 2014;04:260-269. 10.4236/ojrad.2014.43034

[54] Karschnia P , Dono A, Young JS, et al Prognostic evaluation of re-resection for recurrent glioblastoma using the novel rano classification for extent of resection: a report of the rano resect group. Neuro Oncol. 2023;25:1672-1685. 10.1093/neuonc/noad074 PMC10479742 37253096

[55] Wen PY , Weller M, Lee EQ, et al Glioblastoma in adults: a society for neuro-oncology (sno) and European society of neuro-oncology (eano) consensus review on current management and future directions. Neuro Oncol. 2020;22:1073-1113. 10.1093/neuonc/noaa106 PMC7594557 32328653

[56] Stupp R , Mason WP, van den Bent MJ, et al; National Cancer Institute of Canada Clinical Trials Group. Radiotherapy plus concomitant and adjuvant temozolomide for glioblastoma. N Engl J Med. 2005;352:987-996. 10.1056/NEJMoa043330 15758009

[57] Grochans S , Cybulska AM, Simińska D, et al Epidemiology of glioblastoma multiforme-literature review. Cancers (Basel). 2022;14:2412. 10.3390/cancers14102412 PMC9139611 35626018

